# Preconception counselling for low health literate women: an exploration of determinants in the Netherlands

**DOI:** 10.1186/s12978-018-0617-1

**Published:** 2018-11-23

**Authors:** Mirjam P. Fransen, Miriam E. Hopman, Laxsini Murugesu, Ageeth N. Rosman, Sian K. Smith

**Affiliations:** 1 Amsterdam UMC, University of Amsterdam, Department of Public Health, Public Health Research Institute, Meibergdreef 9, Amsterdam, The Netherlands; 2 Rotterdam University of Applied Sciences, School for Healthcare Studies, Department of Master Physician Assistant Midwifery, Rochussenstraat 198, 3015 EK Rotterdam, The Netherlands; 3 The University of New South Wales, Psychosocial Research Group, Prince of Wales Clinical School, Faculty of Medicine, Lowy Research Centre, Sydney, NSW 2052 Australia

**Keywords:** Health literacy, Preconception counselling, Theoretical framework, Determinants, Expert review

## Abstract

**Background:**

Women from lower socioeconomic groups tend to be at greater risk of adverse perinatal outcomes, but are less likely to participate in preconception counselling compared to higher socioeconomic groups. This could be partly because of their limited skills to assess, understand and use health related information in ways that promote and maintain good health (health literacy skills). In this study we explored determinants of participation in preconception counselling among women with low health literacy in The Netherlands.

**Methods:**

Potential determinants of participation in preconception counselling were derived from the literature, and mapped onto a theoretical framework, which was tested for perceived relevance and completeness in an expert review (*n* = 20). The framework was used to prepare face-to-face interviews with women with low health literacy and a wish to conceive (*n* = 139). In the interviews we explored preconception counselling awareness, knowledge, considerations, subjective norms, self-efficacy, attitude, and intention. Linear regression analyses were used to test associations with intention to participate in preconception counselling.

**Results:**

Most women (75%) were unaware of the concept of preconception counselling and the provision of counselling, even if they lived in areas where written invitations had been disseminated. Common considerations for participation were: preparation for pregnancy; perceived lack of information; and problems in a previous pregnancy. Considerations not to participate were mostly related to perceived sufficient knowledge and perceived low risk of perinatal problems. Respondents generally had a positive attitude towards participation in preconception counselling for themselves, and 41% reported that they would participate in preconception counselling.

**Conclusion:**

Women with low health literacy were generally unaware of the concept and provision of preconception counselling, but seemed to be interested in participation. Further research should investigate how to effectively reach and inform this group about preconception counselling. This knowledge is essential for evidence-based development of interventions to increase the accessibility and understanding of preconception counselling.

**Electronic supplementary material:**

The online version of this article (10.1186/s12978-018-0617-1) contains supplementary material, which is available to authorized users.

## Plain English summary

Women from lower socioeconomic groups are more likely to experience adverse perinatal outcomes, but less likely to participate in counselling to prepare for a healthy pregnancy (preconception counselling). These socioeconomic differences could be partly explained by women’s health literacy skills, commonly referred to as the ability to access, understand and use health-related information to promote and maintain good health.). In this study we explored determinants of participation in preconception counselling among women with low health literacy in The Netherlands.

A theoretical framework was tested for perceived relevance and completeness among 20 experts, and then used to prepare face-to-face interviews with 139 women with low health literacy and a wish to conceive.

The interviews showed that most women (75%) were unaware of the concept of preconception counselling and the provision of counselling, even if they lived in areas where written invitations had been disseminated. Common considerations for participation were: preparation for pregnancy; perceived lack of information; and problems in a previous pregnancy. Considerations not to participate were mostly related to perceived sufficient knowledge and perceived low risk of perinatal problems. Respondents generally had a positive attitude towards participation in preconception counselling for themselves, and 41% reported that they would participate in preconception counselling.

In conclusion; women with low health literacy were generally unaware of the concept and provision of preconception counselling, but seemed to be interested in participation. Further research on how to effectively reach and inform this group and to encourage their intention as a catalyst for undertaking preconception counselling is essential to increase the accessibility of preconception counselling.

## Background

Socioeconomic inequalities in adverse perinatal outcomes, such as preterm birth, small for gestational age, low Apgar score, congenital anomalies, and perinatal mortality have been extensively documented. These inequalities occur in both high and low income countries [1–3]. For example, in the Netherlands, fetal mortality is almost twice as high in socially deprived neighborhoods (10.4‰ versus 5.6‰ in non-deprived neighborhoods) [4].

The fact that some of these adverse outcomes are related to modifiable risk factors, such as maternal smoking, alcohol consumption, illicit drug use and inadequate medication use during pregnancy provides a potential opportunity to reduce socioeconomic inequalities through preconception care [5–7]. Preconception care is defined as a set of interventions before conception to decrease the impact of biomedical, behavioural and social risks on a woman’s health, fetal development, and pregnancy outcomes [8]. By the time a woman enters prenatal care, a large part of fetal organ development has taken place [9]. Preconception care is critical for the outcome of the pregnancy, particularly for deprived groups who are more likely to adopt unhealthy behaviours before pregnancy [10]. Preconception interventions can be collective, focusing on the general public, for example by national campaigns. Interventions can also focus on women or couples that are planning a pregnancy, for example by risk assessment, screening and individual counselling [11]. In the Netherlands, individual counselling can be provided through general practices, municipal health services, or midwives and gynaecologists [8]. Preconception care has shown to be effective in improving maternal health behaviour, such as folic acid use, smoking and alcohol cessation, diabetic control and obesity prevention before pregnancy, and preventing congenital disorders [12–15]. Women with a lower socioeconomic background are at greatest risk of complications during and after pregnancy, yet they are least likely to participate in preconception screening, risk assessment and counselling [15–17]. This could be partly related to a woman’s health literacy skills, commonly defined as the ability to assess, understand and use health related information in ways that promote and maintain good health [18]. Health literacy is considered to be an important variable in explaining why socioeconomic differences in health exist [19, 20]. Women with low health literacy are less likely to screen for sexually transmitted diseases, have follow-ups of abnormal test results after cervical cancer screening, and more likely to initiate prenatal care at a later stage of pregnancy [21, 22]. Little is known about the factors associated with participation in preconception counselling, particularly among women with low health literacy. Strategies are needed to improve the accessibility and effectiveness of preconception counselling.

In this study we explored determinants of participation in preconception counselling among women with low health literacy in The Netherlands. The specific objectives were:

- To gain insight into women’s awareness of preconception counselling, and awareness of invitations that were used to recruit women to a pilot program in the Netherlands;
- To assess knowledge, considerations, subjective norm, self-efficacy, attitude, and intention to participate in preconception counselling or not;
- To investigate the extent to which these factors are associated with intention to participate in preconception counselling.

## Methods

### Aim, design and setting

We performed a cross-sectional study to explore factors that play a role in participation in preconception counselling among women with low health literacy.

The study was performed in the Netherlands between April 2014 and November 2016. The study was part of a larger project in which we developed strategies for women with low health literacy and health care providers within the Dutch pilot program ‘Healthy Pregnancy for All (HP4All)’. In the HP4All program, preconception care is delivered in individual counselling that is provided by general practitioners, midwives, or youth health care providers. The HP4All program was initiated in 2011 in specific districts with perinatal mortality and morbidity above the country’s average [8]. Women in the HP4All target municipalities are exposed to letters or flyers, which inform them about preconception counselling and invite them to apply if they have a wish to conceive.

### Research population and recruitment

The research population consisted of 139 women with low health literacy, 72 of them were recruited from a general practice and a youth health care centre in Amsterdam that participated in the HP4All program. This means they were exposed to an invitational letter or flyer for HP4All. The other 67 women were recruited from centres that did not participate in the HP4All program (a youth health care centre, a primary school, and an intermediate vocational education school in Amsterdam, Almere, and Wageningen). This group was not actively invited for preconception counselling, but they could participate in it, as it was offered by all Dutch midwives on request.

Women that were able to communicate verbally in Dutch were personally invited to participate in the study by MH or LM in waiting rooms of the participating centres. We briefly explained the purpose of our study and then asked if they had a wish to conceive pregnancy within 5 years. Those that certainly knew that they did not want to become pregnant in the coming 5 years were excluded. Others were then asked if they had 5–10 min to participate in the Short Assessment of Health Literacy in Dutch (SAHL-D) (see below). Those that had lower health literacy according to the SAHL-D were theninvited for a personal face-to-face structured interview conducted by MH or LM at a time and location that the women preferred. We chose to conduct face-to-face interviews instead of a written or online survey, since we expected that these low health literate women would have difficulty filling in questionnaires, which would lead to unreliable results and a lower participation rate.

### Theoretical framework to guide data collection

#### Von Wagner’s framework for health literacy and health actions

**Fig. 1:**
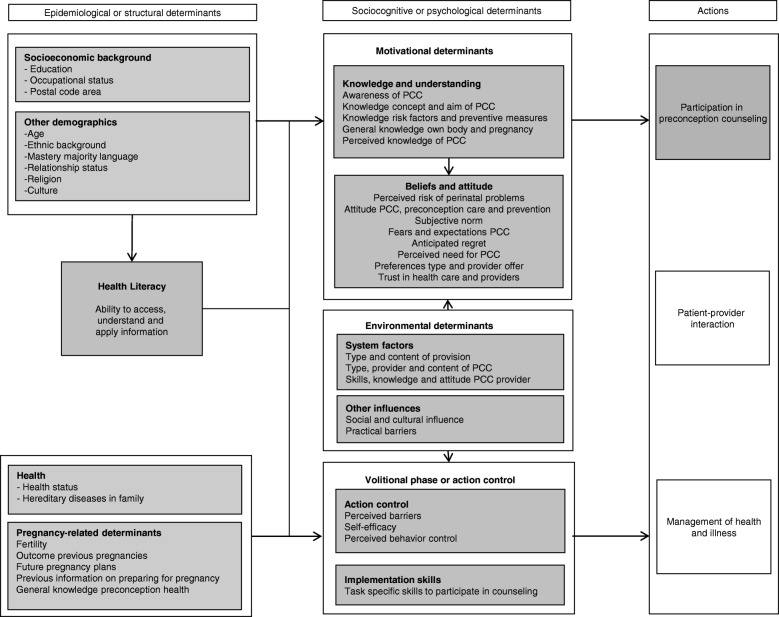
Conceptual framework

The literature search and the development of the questionnaire for the structured interviews was guided by a conceptual theoretical framework (Fig. 1 Conceptual framework). This framework was based on Von Wagner’s framework for health literacy and health actions, that proposes that health outcomes (e.g. preterm birth) are determined by the following actions: access and use of health care (such as preconception counselling); patient-provider interactions; and management of health and illness [23]. These actions are influenced by motivational, environmental, and volitional determinants. Motivational determinants include traditional sociocognitive constructs, such as knowledge, understanding, beliefs, and attitudes. Participation in preconception counseling could for example be influenced by knowing what the counselling involves. Volitional determinants refer to action control, for example self-efficacy, perceived barriers, and implementation skills. Self-efficacy refers to the strength of belief in own ability to complete tasks and reach goals. Implementation skills refer to planning, organizing and task-specific skills i.e. navigational skills to access preconception counselling. Motivational and volitional determinants form a symbiotic relationship with external system factors. These system factors refer to environmental determinants and could include the offer of preconception counselling or out-of-pocket costs for preconception care.

Motivational and volitional determinants are directly affected by health literacy, which in turn is influenced by epidemiological or structural determinants, such as educational attainment level and age.

#### Literature search

In March 2014 Medline was searched using the following search terms: social class, socioeconomic status, social*, socio-economic, low income, education*, education level, health literacy, preconception care, preconception health, preconception counsel*. Reference lists in the included papers were also scrutinized.

In accordance with the conceptual framework described above, we identified the following motivational determinants in the literature: knowledge and awareness of preconception counselling; knowledge of risk factors and preventive measures; attitude; beliefs; and expectations of preconception counselling. Practical barriers and the type of provision were identified as environmental determinants. Volitional determinants that were investigated in the studies included self-efficacy and perceived barriers to participation in preconception counselling. More information on the search and included studies is presented in Additional file 1.

#### Expert review

An expert review was performed to investigate whether the conceptual framework reflected the current experience and knowledge of experts, to ensure a good base for data collection. In total 31 experts were asked by email to fill in an online survey. Of those, 20 participated and filled in an online survey (response rate 64%), consisting of midwives (*n* = 3), researchers (*n* = 9), gynaecologists (*n* = 2), medical students (*n* = 2), advisors (*n* = 2), a nurse (*n* = 1), and a medical doctor (*n* = 1). On average, they had 15 years experience of work (range 1–35 years). The experts confirmed the determinants in the framework and suggested several important additions to complete the framework, including: skills, knowledge and attitude of health care providers (environmental determinants), and task-specific skills to participate in preconception counselling (volitional determinants).

### Measures

The final framework (Fig. 1) was used to prepare data collection in face-to-face interviews. We used the following measures to assess the variables that were derived from the framework:

- *Health literacy* was measured by the Short Assessment of Health Literacy, which was previously adapted and validated for the Dutch situation (SAHL-D) and proved to be a reliable and valid performance-based test to indicate low health literacy in the Netherlands (Cronbach’s alpha = 0.77 for recognition, 0.79 for comprehension and 0.86 for the total score) [24]. Both word recognition and vocabulary are essential for reading comprehension, which is an important element of health literacy [16–18, 24]. During the SAHL-D assessment, respondents were asked to read and pronounce 33 health-related words, which was followed by a multiple choice question about the description of the words. Respondents were assigned one point for each correctly pronounced word and for each correct description. This resulted in a summary score ranging from 0 to 66 points. Following a predefined cut-off point, respondents with a score lower than 55 points were considered as low health literate and could participate in this study [24].
- *Educational level* was based on self-report and categorized using the International Standard Classification of Education, i.e. low (level 0–2: early childhood education; primary education; lower secondary education); intermediate (level 3–5: upper secondary; post-secondary non-tertiary education; short cycle tertiary education); and high (level 6–8: bachelor’s; master’s; doctoral or equivalent level) [25].
- *Ethnic background* was based on the country of birth of the respondents’ parents, in accordance with Statistics Netherlands [26]. Respondents were only considered to be from Dutch ethnic background if both their parents were born in the Netherlands. The respondents were considered to be from another western background, if one parent or both parents were born in Europe, North-America, Indonesia, Japan or Oceania. Respondents were categorized as having a non-western background, if one parent or both parents were born in Turkey, Morocco, Surinam, Netherlands Antilles, Aruba, Africa, Asia (excluding Indonesia and Japan) or Latin-America. If both parents were born in different foreign countries, the mother was the identifier used for ethnicity [26].

To classify *relationship status* respondents were asked whether they were in a relationship or married and, if so, whether they were living together with their partner.

*Wish to conceive* was assessed by asking whether the respondent contemplated pregnancy and in which time frame. Options were ‘in the next two years’, ‘in two to five years’ or ‘yes, but not sure within which time frame’.

*Perinatal experiences* were evaluated by asking respondents whether they had been pregnant before, and if so, whether they had ever experienced unplanned pregnancy (answer options ‘yes’ of ‘no’) or problems during a previous pregnancy (answer options ‘yes’ of ‘no’). The interpretation of experienced problems could encompass feeling uncomfortable and vomiting during the first trimester of pregnancy up to fetal mortality. The interpretation was led by the respondents, because it was used as an evaluation of experiences that could influence women’s choice to participate in counselling and not as a risk assessment.

*Awareness of preconception counselling was* assessed by the question: “Have you heard about preconception counselling?” and “If so, who told you about it?” or “If so, where did you hear or read about it?”. Answers were categorized within predefined sources of information, such as ‘general practitioner’ or ‘newspaper’. Awareness of the written invitation for preconception counselling was defined as having noticed any written invitation (letter or a flyer).

*Considerations to participate in preconception counselling* were assessed by 33 statements on considerations that we derived from the conceptual framework (Fig. 1). For each consideration we asked if this would be a reason to participate or not (answer options ‘yes’ of ‘no’). For eight considerations (e.g. medication use or chronic illness) we assessed the specific denominator by asking if this situation accounted for the respondent (e.g. ‘Do you have a long term illness, disease or disability’ (such as high blood pressure or diabetes)’?’).

*Knowledge on risk factors* for a healthy pregnancy was assessed by six statements that were developed by Temel et al.: pregnancies within a short interval are good for the baby’s health; smoking adversely affects fertility; being underweight or overweight adversely affects fertility; sexually transmitted disease must be treated before pregnancy; all medications from drugstores are safe and can be used during pregnancy; the best moment to start with folic-acid supplementation is when you get pregnant. Response options consisted of ‘true’, ‘false’ or ‘I do not know’ [27]. Answers to the statements were scored as either correct or incorrect (including ‘I do not know’).

*Attitude* towards preconception counselling was measured by a scale of six items (Cronbach’s α = 0.77). Respondents were asked to rate preconception counselling for themselves as good versus bad, comforting versus scary, important versus unimportant, pleasant versus unpleasant, useful versus useless and embarrassing versus something to be proud of. The scale ranged from 6 to 30. For analysis, the total score was divided by the amount of items, which was 6 .

*Intention* to participate in preconception counselling was assessed by asking respondents to rate the likelihood of their participation to preconception counselling before their (next) pregnancy on a 5 point Likert scale ranging from one ‘extremely unlikely’ to five ‘extremely likely’.

*Self-efficacy* was assessed by the statement “I will be able to participate in preconception counselling, if I would like to go” and rated on a similar scale (1 = strongly disagree; 5 = strongly agree).

*Subjective norm* (the perceived social pressure to engage or not to engage in a behaviour) was measured in relation to three categories of important others; friends, family and partner. Respondents were asked for each of the three categories what they assume important others think they should do (− 2 = certainly not participate; + 2 = certainly participate). Then we asked for each assumption whether this would influence their decision to participate (− 2 = not at all; + 2 = very much). After testing internal reliability in our population, we decided to divide subjective norm into two scales: subjective norm related to friends and family members (summary scores ranging from − 20 to + 20) and subjective norm related to their partner (summary scores ranging from − 10 to + 10).

### Statistical analyses

Descriptive statistics were used to summarize background characteristics, awareness, considerations, attitude and intention. Educational and ethnic differences in mean knowledge, mean attitude, and mean intention were analysed by analysis of variance (ANOVA), we did not correct for other variables in this ANOVA, since the primary aim was to assess differences in background variables (educational level and ethnic background). We performed linear regression analyses to test associations with intention to participate in preconception counselling. In the regression analyses we adjusted for potential confounders (educational level and, ethnic background). The covariate was considered as a confounder and left in the model, when it changed the variation in score by 10% or more. We imputed the value of the missing data (‘99’) and included it in the regression analyses, so the sample size will not be reduced. The required sample size for this analysis was based on a power analyses that showed that we would be able to detect a true difference between groups with a low and higher intention to participate in preconception counselling with probability (power) 0,8. The Type I error probability associated with this test of the null hypothesis that the population means of these two groups are equal is 0,05.

## Results

### Background characteristics study population and awareness

A total of 226 women met the inclusion criteria, 87 (38%) did not participate in the interview since they were uninterested in the topic, or perceived the interview to be too personal or too long (response rate 62%). Characteristics of the respondents (*n* = 139) are presented in Table 1. Most respondents had a non-Dutch ethnic background (61%; 45% non-Western and 16% Western, had an intermediate educational level (58%)and lived together with a partner (64%). In total 111 respondents (80%) had been pregnant before, 50% of them reported to have problems during earlier pregnancies, 49% have had an unplanned pregnancy.

**Table 1:** Background characteristics (*n* = 139)

	Mean (SD; range)	N (%)
Age (years)	29.6 (5.6; 18–42)	
Educational level		
Low		10 (7)
Intermediate		81 (58)
High		48 (35)
Ethnic background		
Dutch		54 (39)
Other western (non-Dutch)		23 (16)
Non-western		62 (45)
Health literacy score (SAHL-D)	35 (13; 9–53)	
Relationship status		
Married/Living together with partner		90 (64)
Single/Not living together with partner		50 (36)
Previous pregnancy		
Was pregnant before		111 (80)
Ever had an unplanned pregnancy		54 (49)
Ever had problems in pregnancy		61 (50)
Wish to conceive		
Yes, in next 2 years		41 (30)
Yes, in 2–5 years		61 (44)
Yes, not sure in how many years		37 (26)
Aware of preconception counselling		35 (25)
Subjective norm to participate in preconception counselling^a^		
Subjective norm family/friends (− 8.00–9.00)	−1.72 (3.80; − 8 – 9)	
Subjective norm partner (− 4–5)	0.19 (2.63; − 4 – 5)	

^a^Subjective norm family/friends not applicable to 4 women; subjective norm partner not applicable to 30 women

We included 72 respondents from areas where invitation materials for preconception counselling were disseminated in the HP4All program, 35 of them were recruited in a general practice that disseminated a written invitation by mail, 37 respondents were recruited from a youth health care service where flyers were placed in the waiting room (*n* = 17) or personally handed over (*n* = 20). The other 67 women were included from areas where invitation materials were not standardly provided.

Table 1 further shows that 35 out of 139 respondents (25%) reported that they have heard about preconception counselling before the interview. Of the 72 women that were recruited from the centres that participated in the HP4All program, 22 (31%) were aware of preconception counselling. Of the 67 women that were recruited from other centres, 13 (19%) were aware of preconception counselling.Chi-square tests showed that this difference in awareness was not statistically significant (*p* > 0.05).

Of the 35 women that were recruited in general practices that send out written invitations within HP4All, 11 (31%) remembered receiving the written invitation. Only 1 out of 37 respondents (3%) remembered having received a flyer. Even respondents that were handed over the flyer personally (*n* = 20), did not remember that they received information on preconception counselling. Women that were aware of preconception counselling were slightly less likely to participate in preconception counselling (B-0.22; CI-0.72-0.28).

### Considerations (not) to participate in preconception counselling

Around half of all women (51%) considered participating in preconception counselling because they wanted to prepare for pregnancy (Table 2). This was positively associated with intention to participate (B 1.43; CI 1.06–1.79) (Table 4). Other important considerations for participation were ‘I want information about fertility’ (31%), ‘I have a high risk of perinatal problems’ (29%), and ‘I want to have control over pregnancy’ (29%). For 58% of the 61 women that experienced problems in previous pregnancy (e.g. high blood pressure, nausea, preterm birth), these problems would be a reason to participate in preconception counselling. For 52% of the 33 chronically ill women, their disease would be a reason to participate in counselling. Answers to the open-ended question did not provide other significant categories of considerations.

**Table 2:** Considerations whether or not to participate in preconception counselling (*n* = 139)

Consideration to participate	N (%)	Consideration not to participate	N (%)	Total N
I want to prepare for pregnancy	71 (51)	I already have sufficient knowledge	34 (25)	139
I want information on fertility	43 (31)	I am not interested in counseling in general	30 (22)	139
I have a high risk of perinatal problems	40 (29)	I have a low risk of perinatal problems	23 (17)	139
I want to have control over pregnancy	40 (29)	I am not interested in preconception counseling	17 (12)	139
		I already received info from family and friends	14 (10)	139
I experienced problems in previous pregnancy	35 (58)			61
I have a chronic illness	17 (52)			33
I never received info from GP/Midwife	48 (43)			113
There are hereditary diseases in my family	15 (33)			45
I use medication	9 (30)			30
I ever experienced unplanned pregnancy	14 (26)			52
I have been pregnant before	26 (23)	I have been pregnant before	47 (42)	110 *
I received info from GP/midwife	10 (11)	I already received info from GP/midwife	9 (20)	92

*=number of missing variables

Most frequently mentioned considerations not to participate in preconception counselling in the total population were: ‘I already have sufficient knowledge’ (25%), ‘I am not interested in counselling in general’ (22%), and ‘I have a low risk of perinatal problems’ (17%). Perceived sufficient knowledge and risk perception were negatively associated with intention to participate (Table 4).

In the subgroup of women that had a previous pregnancy (*n* = 110), 42% would not want to participate in counselling, because they had been pregnant before. In answer to the open-ended question, 27% of all respondents mentioned that they would not participate, since they already had sufficient knowledge, 14% mentioned that they would not participate since they preferred another source of information, such as the internet.

### Determinants to participate in preconception counselling

**Fig. 2:**
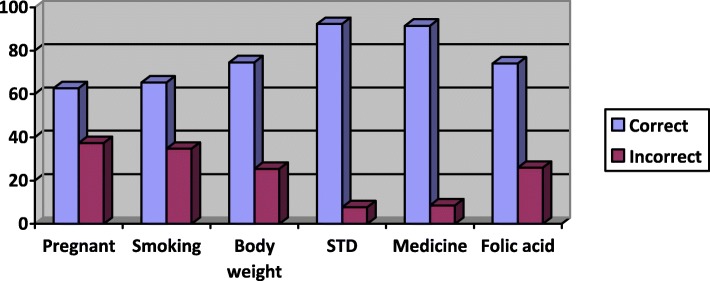
Knowledge risk factors

Figure 2 (Knowledge risk factors) shows the correct and incorrect answers per knowledge item. Lowest scores were obtained for the items ‘Pregnancies within short interval are good for baby’s health’ (63% scored correctly) and ‘Smoking adversely affects fertility’ (65% scored correctly). Highest scores were found for ‘All medications from drugstores are safe and can be used during pregnancy’ (91% scored correctly) and the item ‘Sexually transmitted disease must be treated before pregnancy’ (92% scored correctly). In total 75% of the women scored correctly on the item on body weight, this was 74% for the item on folic acid use.

Table 3 shows total knowledge score (scale 1–6) per educational and ethnic group. Women with a higher educational level scored significantly higher on knowledge than others. Women with a non-Western ethnic background scored significantly lower than women with a Western or Dutch ethnic background. Knowledge was negatively associated with intention (Table 4).

**Table 3:** Knowledge, attitude, self-efficacy and intention preconception counselling (mean;SD)

	Knowledge risk factors (1–6)	Attitude (1–5)	Self-efficacy (1–5)	Intention (1–5)
Total population (*n* = 139)	4.61 (1.25)	3.96 (0.58)	4.19 (0.64)	3.05 (1.30)
Educational level				
Low (*n* = 10)	3.70 (1.49)^1^	3.75 (0.56)	3.80 (0.63)	3.60 (1.71)
Intermediate (*n* = 81)	4.38 (1.18)^2^	3.99 (0.62)	4.26 (0.61)	3.14 (1.27)
High (*n* = 48)	5.17 (1.04)^1 2^	3.96 (0.54)	4.21 (0.59)	2.79 (1.24)
Ethnic background				
Dutch (*n* = 54)	5.00 (1.06)^3^	4.03 (0.65)	4.42 (0.60)^3^	2.80 (1.12)
Other Western (*n* = 23)	5.00 (1.49)^4^	3.93 (0.62)	4.27 (0.55)	3.13 (1.42)
Non-Western (*n* = 62)	4.17 (1.18)^3 4^	3.91 (0.51)	3.97 (0.63)^3^	3.24 (1.39)

23 missings on knowledge

3 missings on attitude

3 missings on self-efficacy

^1^Difference between low and high educational level (*p* < 0.05)

^2^Difference between intermediate and high educational level (*p* < 0.05)

^3^Difference between Dutch and Non-Western ethnic group (*p* < 0.05)

^4^Difference between Western and Non-Western ethnic group (*p* < 0.05)

**Table 4:** Association between determinants and intention to participate in preconception counselling (*n* = 139)

	B (95% CI)	Adjusted B (95% CI)
Awareness (ref unaware)	−0.17 (− 0.67–0.33)	−0.22 (− 0.72–0.28)^a^
Knowledge risk factors (scale 1–6)	−0.15 (− 0.35–0.05)	−0.03 (− 0.25–0.19)^b^
Attitude (scale 1–5)	0.90 (0.55–1.25)	
Self-efficacy (scale 1–5)	− 0.03 (− 0.40–0.34)	0.09 (− 0.29–0.47)^a^
Subjective norm		
Family/friends	0.13 (0.07–0.18)	
Partner	0.22 (0.14–0.30)	
Considerations to participate (ref: no consideration)		
Preparation for pregnancy	1.43(1.06–1.79)	
Perceived lack of information	1.07 (0.54–1.60)	
Problems previous pregnancy	1.01 (0.51–1.51)	
Considerations not to participate (ref: no consideration)		
Perceived sufficient knowledge	−0.30 (−0.49 - -0.12)	
Perceived low risk	−0.48 (− 0.63- -0.29)	−0.53 (− 0.72—0.34)^a^

^a^ adjusted for ethnic background ^b^adjusted for ethnic background and education

Respondents generally had a positive attitude towards participation in preconception counselling for themselves (mean 3.96; scale 1–5; SD 0.58) (Table 3). Mean intention to participate was 3.05 (scale 1–5; SD 1.30), 41% of the respondents reported that they would participate in preconception counselling, 42% would not participate, 17% would perhaps participate. We did not find any significant differences in attitude or intention between educational or ethnic groups.

Mean self-efficacy was 4.19 (scale 1–5; SD 0.64), meaning that respondents generally felt that they were able to participate in preconception counselling if they wanted to (Table 3). Women from non-Western ethnic background scored significantly lower (mean 3.97; SD 0.63) on self-efficacy than women from Dutch ethnic background (mean 4.42; SD 0.60). Higher self-efficacy was positively associated with intention (Table 4).

Subjective norm whether or not to participate in counselling was generally weak. Subjective partner norm was 0.15 (range − 4 -5; SD 2.62), meaning that most women slightly expected that their partner would like them to participate in counselling and that they found his/her opinion slightly important. Women generally perceived a weak subjective norm not to participate in preconception care from family or friends (− 1.76; range − 8-9; SD 3.79). Both subjective norms (partner and family/friends) were positively associated with intention (Table 4).

## Discussion

This study shows that women with low health literacy were generally unaware of the concept and the provision of preconception counselling, but have a positive attitude towards participation in preconception counselling. Intention was quite positive as well, 41% of the respondents reported that they would participate in preconception counselling, 17% would perhaps participate. Most common reasons for participation were to prepare for pregnancy and to gain information about fertility. Considerations not to participate were mostly related to perceived sufficient knowledge, lack of interest in counselling in general, and perceived low risk of perinatal problems. Women generally felt confident to participate in preconception counselling if they wanted to. Subjective norm whether or not to participate was weak. Women from a non-Dutch ethnic background scored significantly lower on knowledge of preconception care and self-efficacy to participate in it.

Only 25% of our respondents had ever heard of preconception counselling. Unawareness is considered to be an important determinant of participation in preconception counselling [28]. We do not exactly know why women in our sample had such a low awareness, even if they lived in an area where invitations for counselling were distributed. In our interviews women explained that they could not remember having seen any letter or flyer. They may not have been exposed to the invitational letter, for example because they did not live on the address that they were registered at their general practice, they might have not opened their mail, or perhaps they did not read the letter because it was too much text for them. As for the flyer, perhaps it was not appealing enough, they did not see the flyer, or simply forgot about it. Unawareness could be related to individual factors such as low health literacy, but could also be affected by factors at provider level. Kransdorf et al. for example reported that young women in college did not discuss reproductive life planning or preconception health with their providers, despite expressing interest in doing so [29]. A qualitative study in Italy found a lack of awareness of preconception health and care, not only among women of childbearing age, but also among midwives, medical doctors and nurses [30].

Although women in our sample were unaware of the concept of preconception counselling, they generally had knowledge on risk factors for a healthy pregnancy. Each item had over 60% of correct scores. Our findings are comparable to the findings of Temel et al. that used the same knowledge items in a study among women in a deprived area in Rotterdam, the Netherlands [27]. They also found that women had poorest knowledge about the adverse effects of smoking on fertility, and that knowledge was lowest among women with a low educational level and a non-Western background. Percentages of correct answers were slightly lower in their sample. We do not know if this was related to lower health literacy levels, since they were not measured in their study. Conrood’s study among a low-income, Mexican American population also found that knowledge on preconception health was high overall, but lower than knowledge among women with a higher economic status in the same region [31].

We found that low health literate women had a positive attitude towards participation in preconception counselling, and that 41% stated that they would like to participate in it. Conrood et al. also found that 43% of the women with a low socioeconomic background in the US was interested to participate in preconception counselling. These levels of interest were similar to those of a higher economic status in the same region [31]. We also did not find any educational or ethnic differences in intention in our group, this might be related to the fact that all our respondents had low health literacy. Temel et al. did find socioeconomic differences in intention to attend preconception counselling [32]. Those with a lower socioeconomic background had a higher intention to participate. However, Temel et al. did not measure intention to personally participate, but an overall attitude towards preconception care, measured by the statement ‘A woman who wishes to become pregnant should consult a GP or midwife before she tries to become pregnant’.

In contrast to most other studies, women in our study reported more considerations in favour of counselling, than considerations against counselling [28]. An important reason to participate, besides preparing well for pregnancy, was to have information on fertility. In their systematic review Poels et al. found that “believing in the benefits” and “availability of preconception counselling” were the most frequently identified facilitators for counselling use. Considerations against counselling did not seem to differ that much from those that were found in studies among women in the general population. Poels et al. also found that “Not (fully) planning pregnancy”, “perceived absence of risks”, “lack of awareness”, and “ adverse pregnancy experiences” were the most frequently identified barriers to participate in preconception counselling [28].

We found weak subjective norms regarding participation in counselling. This is not in coherence with other studies, but this discrepancy may be related to differences in measurement [27, 31]. For example, in focus group interviews Afro- American women explained how social factors influenced participation in preconception counselling, but they were not quantified [31]. Temel et al. quantitatively assessed subjective norm and also found that partners were the most important social influence in deciding whether or not to participate in counselling. However, they only assessed perceived importance of the opinion of important others, and not what respondents thought others would advise them to do [27]. Most of our respondents also rated their partner’s norm to be important, but the second component of subjective norm (perceived partner’s opinion on participation) was mostly scored as neutral, resulting in a weak subjective norm to participate.

Almost half of the respondents (49%) in our study have had at least one unplanned pregnancy. This is far above the national prevalence of 20% unplanned pregnancies in the Netherlands [33]. Previous studies also indicated that women with low health literacy more often have unplanned pregnancies than others [22]. This suggests that the current concept of preconception care is limited for women with low health literacy and confirms the importance of reaching this group of women on time.

This study has several strengths and limitations. A strength of this study is that it provides insight in women’s considerations and other factors that could play a role in participation in preconception care. We used the SAHL-D to include women with low health literacy. This performance-based test is frequently used by others and validated in the Dutch context [24]. However, it only measures skills to read and understand and not more advanced skills like appraising or applying information, or context specific skills that are needed to participate in preconception counselling. We only included women with low health literacy. This enabled us to collect data for intervention development for this specific population, but meant we did not have a higher health literacy group to compare our findings with. More than half of our study population lived in an area where invitations for preconception care were sent out, this potentially could have led to bias in our outcome measures. However, only 17% of the women remembered that they received an invitation. Awareness of preconception care and knowledge of risk factors did not significantly differ between women who did not receive an invitation. We performed structured face-to-face interviews to increase our response and to ensure reliable data collection. Although we emphasized our neutral role as researchers, it could be possible that women provided social desirable answers. Another limitation is that the time frame chosen for ‘wish to conceive’ was quit broad (within 5 years). We chose this time frame to exclude women that certainly knew that they did not want to become pregnant in the coming 5 years, and to include those that have a certain interest in the topic (and may become pregnant, either planned or unplanned in the future). A last limitation is that 38% of the women that met the inclusion criteria did not participate in the study. This could have led to sampling bias, and an underestimation of our findings, since low interest in the topic was one of the reasons for them not to participate in the study.

## Conclusions

Women with low health literacy are generally unaware of the concept and provision of preconception counselling, but seem to be interested in participation. This study emphasises the need for recruitment strategies that are tailored to their skills and daily lives. These strategies should raise awareness of the concept of preconception health and preconception care, and explain the benefits and importance of it. Strategies should be applied in time and to all individuals, not just women that are planning a pregnancy. Our findings raise questions about the conditions and efficacy of general advertisement for this group. Entertainment education strategies, applied games, or new media offer promising perspectives to raise awareness. Other options are to provide counselling opportunistically e.g. during other health care visits. Further research should investigate to what extent such strategies are feasible and effective for individuals with low as well as those with adequate health literacy levels.

## Additional file

Additional file 1: Literature search. (DOC 48 kb)

## Abbreviations

ANOVA: Analyses of variance
HP4All: Healthy Pregnancy for All
SAHL-D: Short Assessment of Health Literacy in Dutch
