# Supplementary material for: Preconception counselling for low health literate women: an exploration of determinants in the Netherlands
Source: Reprod Health. 2018 Nov 23;15:192. doi: 10.1186/s12978-018-0617-1 (PMC6251122; doi:10.1186/s12978-018-0617-1)
Supplement: Supplementary file 1 — Literature search. (DOC 48 kb) [file 12978_2018_617_MOESM1_ESM.doc]

For attachment I: Literature search

**Search strategy and selection**

We conducted a literature study to specify and verify Von Wagner’s framework for participation in preconception counseling. We aimed to identify studies on the role of health literacy and other determinants in participation of low SES groups.

In March 2014 Medline was searched using the following search terms: Social Class, Socioeconomic Status, social*, socio-economic, low income, education*, education level, health literacy, preconception care, preconception health, preconception counsel*. Reference lists in the included papers were also scrutinized.

Original qualitative or quantitative studies among individuals with low SES were included if they reported on participation in preconception counseling in any form (personal, group, digital) and/or variables that could influence participation in preconception counseling (potential determinants). Studies were excluded if they only investigated use of screening, risk assessment, food supplements or medication (i.e. without counseling), or if the studied preconception counseling was aimed at a specific population (e.g. women with diabetes) and not the general public. Studies were also excluded if they did not report in English or if the study was performed in a non-Western country. Non-peer reviewed papers, literature reviews, abstracts, dissertations, single case reports, editorials, commentaries, conference abstracts, and non-research papers were excluded as well.

Figure 1 demonstrates the selection of papers. The literature search was performed by MH and JS and yielded 313 papers; 32 papers were selected based on title and abstract by MH, JS and MF; after reading full text, 8 papers were selected for the review by MH, JS and MF. An additional two papers were included after searching the references of the selected papers. In total 10 original studies describing the participation in preconception counseling by individuals with low SES or socioeconomic differences in participation were included.

**Data extraction and quality assessment**

For each paper, MH and JS extracted and summarized relevant data on study design, the aim of the study, type of preconception counseling, study population, SES indicator (e.g. educational attainment level, postal code area, household income), determinants of participation (motivational, volitional and environmental determinants, health literacy, and background variables), and main findings. Two authors (MH and MF) independently assessed the quality of each study and then discussed the results to reach consensus. The assessment of the methodological quality of the quantitative studies was guided by a selection of seven predefined criteria, informed by the Dutch Cochrane Centre (21). These criteria refer to a clear description of the study design, data collection methods, study population, setting and measures, and adequate control for important confounders. The quality of included qualitative studies was evaluated by a checklist for qualitative studies of the Dutch Cochrane Centre. This checklist contains seven components: Relevant and adequate research question; appropriate method for data collection; adequate sampling; verifiable results; adequate analyses; fundamental idea behind research is clear; conclusion fits qualitative character of research.

**Figure 1 Flowdiagram of studies included in the review**

Publications retrieved from Medline search (n=313)

Excluded based on titles/abstract (n=281)

Full-text articles reviewed (n=32)

Excluded based on full text (n=24)

Added after searching references of selected articles (n=2)

Original studies included (n=10)
